# Personal Genome Sequencing in Ostensibly Healthy Individuals and the PeopleSeq Consortium

**DOI:** 10.3390/jpm6020014

**Published:** 2016-03-25

**Authors:** Michael D. Linderman, Daiva E. Nielsen, Robert C. Green

**Affiliations:** 1 Department of Genetics and Genomic Sciences, Icahn School of Medicine at Mount Sinai, New York, NY 10029, USA; 2 Division of Genetics, Department of Medicine, Brigham and Women’s Hospital, Boston, MA 02115, USA; dnielsen@genetics.med.harvard.edu; 3 Harvard Medical School, Boston, MA 02115, USA; 4 Broad Institute of MIT and Harvard, Cambridge, MA 02142, USA; 5 Partners Personalized Medicine, Cambridge, MA 02139, USA

**Keywords:** personal genome sequencing, return of results, population screening, genomics

## Abstract

Thousands of ostensibly healthy individuals have had their exome or genome sequenced, but a much smaller number of these individuals have received any personal genomic results from that sequencing. We term those projects in which ostensibly healthy participants can receive sequencing-derived genetic findings and may also have access to their genomic data as participatory predispositional personal genome sequencing (PPGS). Here we are focused on genome sequencing applied in a pre-symptomatic context and so define PPGS to exclude diagnostic genome sequencing intended to identify the molecular cause of suspected or diagnosed genetic disease. In this report we describe the design of completed and underway PPGS projects, briefly summarize the results reported to date and introduce the PeopleSeq Consortium, a newly formed collaboration of PPGS projects designed to collect much-needed longitudinal outcome data.

## 1. Introduction

Many thousands of ostensibly healthy individuals have had their exome or genome sequenced, but a much smaller number of these individuals have received any personal genomic results derived from that sequencing. Those who have are among the first to test the role for personal genome sequencing (PGS) in effecting more personalized and preventative medicine. In this report, we term those projects in which ostensibly healthy participants can receive sequencing-derived genetic findings and sometimes have access to their genome sequence data as participatory predispositional personal genome sequencing (PPGS). Here we describe the motivation, design and results-to-date from this important and growing class of translational genomics projects.

Our focus in this report is predispositional (or pre-symptomatic) genomic screening to identify disease risk or other personally useful information. Thus we exclude diagnostic genome sequencing intended to identify the molecular cause of suspected or diagnosed genetic disease in the participant or a family member. We are further exclusively focused on sequencing technologies (as opposed to genotyping) that can also identify novel genetic variation.

Predispositional screening is being approached from several directions and perspectives, including medical science, health policy and commercial innovation. For some, PPGS for risk assessment for actionable medical conditions is the natural extension to healthy individuals of opportunistic screening for incidental findings in diagnostic testing [1]. For others, direct-to-consumer (DTC) sequencing is the inevitable next step after DTC genotyping. And for still others, there is an expectation that existing public health screening programs, such as state-mandated newborn screening, will be extended to more comprehensive genome sequencing technologies. Underlying all of these is the narrative that today’s reactive, symptom-based health care system must eventually give way to predictive, preventive, personalized and participatory models of wellness and health maintenance.

The Personal Genome Project (PGP) [2], initiated in 2005 to collect and make publicly available comprehensive personal health information and genomic data, was arguably the first participatory PPGS project. At the time, however, genome sequencing was prohibitively expensive and so the first PGP whole genome was not published until several years later [3]. The first participatory personal whole genome sequences were published in 2007 and 2008, for J. Craig Venter [4] and James Watson [5] respectively. These efforts were primarily technology demonstrations, but also served as proofs-of-concept for predispositional genomic risk assessment. Additional individual or nuclear family participatory genomes have been published since to demonstrate new sequencing technologies [3,6], comprehensive individual [7] and family-based [8] risk assessment and personal “panomics” [9]. The rapidly dropping cost of whole exome and genome sequencing (GS) in the last five years now makes it possible to execute large-scale PPGS projects that systematically examine the potential benefits and harms of this technology, and a number of additional PPGS projects have been initiated (Table 1).

In most of these projects, the goal for PPGS is to provide genetically informed predictions of disease risk, medication safety/efficacy and other information that enables recipients to take a more personalized and preventive approach to their health and wellbeing. And there can also be ethical and other non-health-related motivations to return genomic data to project participants independent of any potential health benefits. But at this moment in time, the use of PGS for population screening in ostensibly healthy individuals is controversial, with the preponderance of experts and professional organizations recommending caution and calling for more research, given large gaps in evidence around penetrance, clinical validity and clinical utility [10,11,12,13,14,15,16,17]. However, there is also a convergence of forces that are accelerating PPGS for persons who would not meet the current criteria for diagnostic sequencing. These forces include the broad social empowerment of individuals and disintermediation of traditional authority in health care, and the launch of governmental and commercial sequencing enterprises that hope to generate discoveries that will accelerate pharmaceutical development. Above all, there is a powerful, but as yet unproven, narrative that learning more information about one’s health risks in virtually any form will be beneficial.

Since so few individuals have received PPGS results to date, and most who have did so recently, little is known about the near and long-term outcomes of PPGS. Many of the projects described herein were initiated to improve our understanding in this area. We begin by reviewing the potential benefits and harms of participatory PPGS. We then describe previous and ongoing participatory PPGS projects, briefly summarizing the results reported to date. Finally, we introduce a newly formed collaboration, the PeopleSeq Consortium, designed to collect longitudinal outcome data across several of the cohorts described herein.

## 2. Potential Benefits and Harms of PPGS

Here we briefly describe some of the potential benefits and harms of PPGS, and point the interested reader to relevant chapters [10], reviews [16,18] and the cited articles below.

The stated goal of most PPGS programs is to provide ostensibly healthy individuals with genetically informed predictions about their risk of developing disease and information about medication safety/efficacy that could inform preemptive clinical management and disease prevention. To the extent that this goal may be achievable, PPGS would likely be most effective for identifying “outlier” individuals with rare “high-effect” variants [19], such as those associated with increased risk of cancer or certain cardiac events, or with pharmacogenomic (PGx) variants that would make the use of specific drugs unsafe or ineffective [20]. However, there is also evidence to suggest that some recipients of risk predictions for common polygenic disease make positive behavioral and lifestyle changes and can become more engaged with their care [21,22,23,24]. And when combined with other data modalities (or panomics), PPGS may inform a more quantitative estimate of wellness [25].

There may also be ethical or non-health-related motivations for designing participatory genomics projects [26,27,28,29]. For some it is a moral imperative to return research results to participants who gave their time, specimen and in many cases tax dollars, to support that work. The increased engagement with research participants required when returning results may ultimately benefit the project, investigators and the scientific community at large. Or in the specific case of the PGP, the return of genomic data may be inherent in a project designed to make its data public and in which the investigators do not believe that they can guarantee participants’ anonymity (and thus do not attempt to do so) [30].

There are also numerous concerns when returning PGS results to participants, particularly with the increased scope and complexity of GS [10,11,12,13,14,15,16,31]. The results could be distressing without any corresponding clinical or personal benefit. False-positive or uncertain results could prompt unnecessary or non-beneficent follow-up care. Probabilistic results that are misinterpreted as deterministic could motivate recipients to seek expensive or invasive care for conditions that they would ultimately never have developed. This unnecessary care could consume finite resources and could create iatrogenic harms. Alternately recipients could be falsely reassured by their negative results or become fatalistic in response to positive results and thus not seek the care they need. Participants may be concerned about how their genetic information may be used by others, including to identify them against their wishes or to make insurance underwriting decisions.

As defined here PPGS is a form of population screening, and will likely be judged by similar criteria as other genetic and public health screening tests [13,14,15,16,19,32,33]. At present there is very limited data on the medical outcomes of sequencing unselected ostensibly healthy individuals. Some of the potential health-related benefits are inferred, for example the possibility of detecting monogenic cancer susceptibility variants that would allow for earlier or more comprehensive screening. Similarly, at present, the actuality and severity of the potential harms, and costs to the healthcare systems, are largely unknown.

## 3. PPGS Projects

Table 1 lists attributes of participatory PPGS projects. These projects were selected according to criteria described previously: sequencing projects that return health-relevant genetic results to pre-symptomatic individuals. We have excluded projects that exclusively enroll individuals with suspected genetic disease, such as Genomics England’s 100,000 Genomes Project; projects that do not return results to participants, such as the U.S. Department of Veterans Affairs’ Million Veteran Program; and projects that only return carrier status. Although we attempted to assemble a comprehensive list, given the rapid growth in this area, we expect some projects may have been missed. For example, as this article goes to press we are aware of PPGS services available through Arivale [34], HealthNucleus [35] and Veritas Genetics [36], and several “personal omics profile” projects under development, such as the Google Baseline Study, that may or may not include a participatory PPGS element.

As shown in Table 1, there are differences among the projects in every attribute, reflecting each project’s specific context (clinical, research, educational, commercial) and aims. Two projects that are particular outliers are the “Practical Analysis of Your Personal Genome” (PAPG) course and the BabySeq Project. In PAPG, students are offered the opportunity to obtain and analyze their own whole genome sequence as part of the course. The students perform the analysis themselves and so no findings are “returned”, although the students can generate similar results for themselves [37]. The BabySeq Project examines PPGS in healthy newborns, in contrast to the other projects, which are exclusively or primarily focused on adults [38].

Although by definition all of the listed projects return genomic results to participants in some way, for some of the projects doing so is an intrinsic purpose of the project. For other projects, results are only returned to a subset of participants in specific situations, for example, when clinically significant variants are detected. The HealthSeq Study is an example of the former, while the ClinSeq Study is an example of the latter. In PGP-Harvard, the return of the genomic data to the participant is not necessarily an intrinsic purpose of the project, but is an explicit and necessary step in the protocol. To enable informed sharing, participants are provided private access to their genomic data and a filtered list of variants with literature annotations to review prior to either sharing their data publicly or withdrawing from the study.

All of the projects return monogenic disease-associated variants in some form, with some projects also reporting PGx and common polygenic disease risk findings. The HealthSeq Study additionally provided some physical traits (such as bitter taster) and ancestry findings. Within these broad categories there can be substantial differences among the PPGS projects in the scope of information returned. For example some projects will only return monogenic disease variants in a limited set of genes (e.g., 76 genes for Geisinger MyCode), while others will return any monogenic disease variants that are found in a much larger set of disease-associated genes (e.g., over 7000 genes for the MedSeq Project). Further each project uses a different interpretation protocol for determining pathogenicity. These differences make it challenging to compare population findings, e.g., average number of “reportable” variants per participant, between projects.

Project participants in each of these cohorts are recruited in a variety of settings and from a variety of healthy or diseased communities. A key distinction is whether the PGS is performed inside or outside the context of an existing clinical relationship, such as a participant’s current primary care or specialist provider (e.g., MedSeq and BabySeq Projects), or outside of a clinical setting entirely (e.g., PGP or the 23andMe Exome Pilot). Participants recruited by their physician might have different pre-test motivations and concerns, as well as different post-test outcomes, than those who self-select by responding to advertisement or other opportunities or who interact with a company or project team outside their existing clinical relationships. Those projects performed in the context of an existing clinical relationship, such as the MedSeq Project and the Illumina TruGenome**™** Predisposition Screen, are also typically implemented as a clinical test whose results will, or could, be directly incorporated into the participant’s/patient’s health record. Projects built around a research test may or may not offer clinical confirmatory testing as part of the project itself. Sometimes, participants are directed to seek out follow-up clinical testing on their own before acting on their results.

All of the projects described in Table 1 obtain informed consent prior to sequencing, with most of them incorporating an Institutional Review Board (IRB)-approved protocol and consent process. However, a few projects, such as the Illumina TruGenome**™** Predisposition Screen, are offered as physician-ordered commercial testing products and in these, only a clinical consent is required. The consent process may have multiple stages; for example in some research projects, the participant may complete an initial shorter consent prior to taking a questionnaire or other preparatory task and then a longer consent prior to sequencing. Some, but not all, projects incorporate “traditional” in-person pre-test genetic counseling [56], including collecting family and medical history, risk assessment and comprehensive education, either as part of or in addition to the informed consent process. For the physician-ordered tests, the ordering physician is typically responsible for pre-test genetic counseling.

During the consent and pre-test counseling process some projects provide an opt-out menu [57], either for all results, or for additional results beyond a minimum set. This opt-out may be structured by category of information, such as actionable monogenic disease variants and PGx; level of confidence, such as variants of uncertain significance; or disease area. In projects that return a restricted set of findings it may be possible to enumerate the set of potential findings during the consent/counseling process. However, many projects perform a true genome-wide analysis in which the potential findings cannot be known *a priori*. In that context any pre-test opt-out is necessarily discussed at a coarser granularity, for example the exclusion might entail “neurodegenerative diseases” as opposed to Alzheimer disease. In some projects there is also an additional opportunity to opt-out post-test prior to or during the return of results, either as a formal component of the protocol’s iterative consent model [58] or informally in the discussion with the reporting provider.

Many of the reviewed projects produce a report document with some or all of the following: a description of the test, relevant quality/coverage data, genomic findings and recommendations. The reporting provider often communicates the results verbally to the participant, and the report itself may or may not also be provided to the participant. Depending on the specific aims of the project this report may be designed for a medical provider or the lay participant. Some projects additionally or exclusively provide results in electronic format, such as through an app (Illumina UYG) or as an online resource (PGP-Harvard).

## 4. Results to Date

At present most of the PPGS projects described in this report are still underway and so only a few have published findings. In this section, we briefly summarize the published results to date.

### 4.1. Participant Motivations, Concerns and Intentions

Multiple projects have reported results from pre- and post-test questionnaires and interviews assessing participants’ motivations, concerns, expectations and intentions [42,48,59,60,61,62,63].

Commonly reported motivations for enrolling included: to learn health-related information, general curiosity, and, where relevant, contribute to research or professional development. Not all PPGS projects are research studies; Illumina UYG, which incorporates the Illumina TruGenome**™** Predisposition Screen, is designed in part as an educational program targeted at physicians and life-science professionals interested in learning about genome sequencing. More generally, PPGS cohorts are enriched for highly educated early adopters with higher socio-economic status. And it seems that participants’ curiosity, about themselves and about the technology, is a common motivation alongside the desire to obtain personally-relevant health-related information and altruism motivations also reported in other translational research studies [48,59]. Unlike many of the PPGS projects reviewed, the HealthSeq Study also reported non-health-related information, such as ancestry. More than a third of HealthSeq participants reported that obtaining ancestry information was a “very important” motivation for enrolling [48].

Reported concerns expressed by participants include potential psychological distress and the privacy of their data, particularly privacy around exposure to insurance discrimination. Those HealthSeq participants who provided additional detail about their privacy concerns specifically cited insurance related concerns [48]. In the MedSeq Project, 28% of prospective participants who declined to enroll cited insurance discrimination as their primary reason [63,64]. As noted by the study investigators, it is unclear whether these refusals reflect that the Genetic Information Non-Discrimination Act (GINA) was insufficiently understood, or whether it was fully understood and the concerns were about unprotected classes of insurance, such as life or long-term care insurance.

Across the PPGS projects that reported participant intentions, ClinSeq, Baylor YPO, HealthSeq, and a single Illumina UYG event at the University of Minnesota (hereafter termed the UMN UYG cohort), almost all participants wanted to receive results and most, but not all participants, wanted all available results. For example 294 of 311 ClinSeq participants reported that they wished to learn results with none reporting that they did not wish to learn results [60] and 33 of 35 HealthSeq participants (94%) wanted to receive all personal GS results available [48]. Although there was less desire to receive certain classes of results, such as variants of unknown significance, the reductions were minimal. Enthusiasm to receive nearly all available results was high initially and largely unchanged after completing informed consent and pre-test counseling.

Since there is not yet evidence that PPGS can improve their health, participants’ enthusiasm for receiving PGS results and strong motivation to apply that information to improve their health suggests that participants may have unrealistically high expectations for the utility of PPGS results [60]. For example, in a survey of the UMN UYG participants, 5 of 17 participants completing the post-test survey reported that they were surprised by the lack of findings [61]. These results suggest that, even in a cohort in which all participants’ self-reported at least moderate familiarity with GS, there is a mismatch of expectations around the medical utility of the results.

### 4.2. Monogenic Disease Variant Burden and Outcomes

A number of projects have published descriptions of the monogenic disease-associated variants reported to project participants [42,51,65,66,67]. Each project uses a different variant filtering and interpretation workflow and reports the number and type of variants differently making comparisons difficult. For the same reasons, these results cannot be directly compared to analyses of incidental findings variants in anonymized cohorts, such as the Exome Sequencing Project [68,69].

Only a subset of the reviewed projects include longitudinal surveillance or follow-up phenotyping, either as part of the project itself or through structure or secondary self-report by the participant. And many of those projects that do include such follow-up are still ongoing. To date PGP-Harvard has reported examples of “true positive” findings and uncertain findings that prompted ultimately negative diagnostic testing, but not in a systematic manner [70]. The Baylor YPO identified 23 “disease associations” where the variant was said to be informative for the participant’s family or medical history [42]. The ClinSeq study recently reported identifying putative loss-of-function variants likely to cause a phenotype in heterozygotes in 103 of 951 participants [66]. Seventy-nine of the 103 participants were evaluated, of whom, 34 had “findings or family history that could be attributed to the variant”. Based on these results, the ClinSeq investigators predict that 1 in 30 unselected individuals would have such a variant. In a separate study of 29 ClinSeq participants who received genetic findings, 72% reported sharing their result with at least one health-care provider, however only a minority (31%) reported making any changes to their health-care in response to their results [71]. The Stanford GMAP reported on recommended and actual follow-up by at least one participant (risk reducing surgery and intensified screening prompted by a risk allele for hereditary breast and ovarian cancer) [51]. Two self-reported instances of follow-up were described in the UMN UYG cohort: a blood test to diagnose a potential enzyme deficiency, and notification of physicians about potential risk for malignant hypothermia prior to surgery [61]. More generally in that cohort, 7 of 28 participants reported receiving a medically actionable finding, but it was unclear exactly what kinds of variants respondents were describing.

### 4.3. Psychosocial and Other Behavioral Outcomes

As many PPGS projects are ongoing, there have been relatively few published analyses yet of psychosocial or other behavioral outcomes of PPGS. In the Baylor YPO, of the 42 participants who responded to a post-test survey (of 81 original participants) 97% agreed or strongly agreed that “they were glad they decided to participate in the study” and 25% reported taking an action of some kind after receiving their results (defined as undergoing follow-up tests, seeing a specialist, or changing insurance, medication, exercise, vitamins/supplements) [42]. In the UMN UYG cohort, most respondents did not regret their decision to participate immediately post testing or 3 months later [61]. In a mixed-methods study of 29 ClinSeq participants who received genetic findings, respondents generally scored high on the Positive Experience subscale and low on the Distress subscale of the MICRA instrument [71]. Common themes in the qualitative component were increased self-awareness and vigilance in response to their genetic results. In the slightly different context of the PAPG Course, students who analyzed their own genome generally reported high decision satisfaction, low decision regret and low test related distress on standardized measures [72]. One exception was a student who experienced a brief period of distress after finding a variant of unknown significance in a gene associated with Brugada Syndrome, an adult-onset cardiac phenotype, which they ultimately determined to be benign.

## 5. Discussion

The application of personal genome sequencing for predispositional population screening for genetic disease risk is perceived by many to be an inevitable next step for genomic medicine. With that expectation in mind, many of the projects described in this review were designed to systematically test the role for PPGS in enabling preemptive clinical management and disease prevention.

In contrast to diagnostic genomic testing, PPGS is not intended to identify the molecular cause for suspected or diagnosed genetic disease, but instead to screen for disease associated variants in the absence of specific symptoms or family history. In this respect there are some similarities between PPGS and the return of incidental or secondary findings during diagnostic testing [1,10]. By some definitions all PPGS findings could be described as incidental or at least unanticipated. However, this parallel does not reflect the very different contexts in which PPGS and diagnostic sequencing take place. For example, in the ACMG recommendations on incidental findings, opportunistic screening for incidental findings was distinguished from population screening by noting that in the former, patients were already undergoing counseling and likely had medical experts available who could contextualize any secondary findings [73,74]. Thus, while concerns were raised about the ACMG recommendations [75] most sequencing laboratories have adopted the position of routinely offering at least this degree of secondary findings.

Although we describe PPGS participants as healthy we should not presume that they are all equally unaffected by personal medical conditions or by a family history that might be suggestive for genetic disease. Everyone falls somewhere along the spectrum of healthy to chronic conditions to acute illness, hence the description “ostensibly healthy” for PPGS participants. And PPGS cohorts, particularly the early adopters, may be enriched for individuals with a baseline perception of elevated risk or subtle symptoms or family histories that do not cross the threshold for initiating diagnostic testing, but still concerns them. For example, the study authors suggest that the high number of disease-associated variants detected in the Baylor YPO cohort likely reflects the self-selection into the study of individuals with an increased prior for genetic disease or disease risk [42]. And in the HealthSeq cohort, 5 of 35 participants reported being motivated by known or suspected personal history of disease, and 12 of 35 by known family history of disease [48].

The goal for PPGS is to provide recipients with genetically informed predictions of disease risk and medication safety/efficacy that can inform their medical and personal decision-making. There also may be ethical or practical motivations for returning genomic results to research participants independent of any potential health-related benefits. However, there are significant challenges to be addressed. Implementing PPGS involves all the same interpretation challenges as diagnostic testing but with generally increased scope and without the benefit of a strong prior probability for genetic disease [13,14,15,16,19,32,33]. As more healthy individuals with detailed phenotypes are sequenced, our understanding of variant penetrance and disease expressivity, which are currently biased by ascertainment in affected individuals, will change. Expanding the number of people undergoing genomic screening will require a corresponding increase, through improved training and education, in the number of clinical professionals prepared to implement and apply these tests in a predispositional setting [37,76,77,78]. Further, as the expectations around the return of results by genomics research studies evolve, new guidelines and best practices are needed to ensure research findings are analytically and clinically valid and returned to participants in an appropriate and legal manner [79].

There is intense and evolving debate about if and how to most effectively and appropriately apply sequencing technologies for predispositional population screening [10,11,12,13,14,15,16,19,32,33,80]. The promise of genome sequencing is the ability to implement a single genomic test that can detect both known and novel disease-associated variants for a variety of medical uses. This more comprehensive testing concept combines multiple, very different, applications: screening for highly penetrant monogenic disorders directly relevant to the recipient’s health, carrier screening, screening for common polygenic disease susceptibility, and screening for medication safety/efficacy. Each of these applications will have different clinical validity and utility. We note that the projects described here have made a variety of choices about the appropriate (and feasible) scope for PPGS, with some offering all of the above results, while others are more narrowly targeted. We should expect, though, that there will be constant pressure to expand the breadth of results reported in PPGS projects.

At present the benefits and harms of PPGS are mostly speculative, as there are little data available about both short and long-term PPGS outcomes and costs [81]. Although there are now more than 1000 ostensibly healthy individuals who have received PGS findings, most have not been systematically followed in a coordinated fashion to measure clinical and psychosocial outcomes.

## 6. A Next Step: The PeopleSeq Consortium

The Personal Genome Sequencing Outcomes (PeopleSeq) Consortium is the first systematic large-scale longitudinal study of the outcomes of PPGS. The PeopleSeq Consortium is a collaborative effort of multiple participatory PPGS projects to collect short and long-term medical, behavioral and economic outcomes data across different projects using a common set of pre and post-test online survey instruments. Currently participating projects are indicated in Table 1. Table 2 summarizes the categories of data being collected by the PeopleSeq Consortium.

Participants in the PeopleSeq Consortium are invited to complete a baseline survey prior to having their genome sequencing results disclosed. A post-disclosure survey is administered 2–3 months after participants receive their results to measure short-term outcomes. We are currently developing a follow-up survey to measure longer-term outcomes that will be completed by participants annually for the duration of the investigation. Long-term follow-up is important because the medical, behavioral and economic impacts of undergoing PPGS will likely play out over many years, with participants potentially using their genome sequencing data as an ongoing resource to make health, financial and other decisions. As the PeopleSeq Consortium progresses, we expect to capture data on changes to participants’ health status, including any evolution of their family histories; and what impact, if any, their genome sequencing data might have on their clinical management and disease prevention strategies. The PeopleSeq Consortium will provide much needed data on the benefits, harms, and cost-effectiveness of PPGS, which are currently not known, but of great interest to clinicians, researchers and policy makers.

To date, nearly 600 individuals have been invited to participate in the PeopleSeq Study from three cohorts, Illumina UYG, PGP-Harvard and HealthSeq, with approximately half of all individuals contacted enrolling. Additional cohorts have recently joined the Consortium (see those identified in Table 1) and data from these groups will be added over the coming year. Preliminary data from early responses has already provided insights on the relationship between motivations to pursue PPGS and perceptions of utility. For example, approximately 20% of PeopleSeq participants report seeking out PPGS due to specific concerns about their family history, and those that cite this as one of their motivations are more likely to report learning something from their genomic results that they believe will improve their health compared to those who did not report family history concerns [82]. These preliminary results support the notion, described in a previous section, that some ostensibly healthy adults seek out PPGS because of specific medical concerns.

As the PeopleSeq Consortium grows, we will further compare participant outcomes from different projects to illuminate the impacts of different approaches to pre-test education and consent, genome interpretation and reporting. The different project designs represent perturbations that can help test the impact of different protocols and identify best practices for PPGS.

## 7. Conclusions

This report summarizes projects that are investigating the impacts of participatory predispositional personal genome sequencing in ostensibly healthy adults. Such projects are one component of the broader research enterprise conducting experiments and demonstration projects around the return of PGS results within and outside of the medical system. These initiatives will add to the data being collected around the use of GS as a screening tool. Refining sequencing standards and methods for variant interpretation [83,84,85], the sharing of genomic information [86], and generating evidence for clinical utility and cost-effectiveness [87] of PPGS will take some time, and in the absence of these data, returning genomic results to ostensibly healthy individuals and their families will remain a subject of considerable controversy.

## Figures and Tables

**Table 1:** Summary of participatory PPGS projects. Projects with a “*” are current members of the PeopleSeq Consortium. Numbers were collected in October 2015 to January 2016. Laboratory Report with Signout indicates if recipients receive a report “signed out” by a clinical professional, such as medical or laboratory geneticist; projects with “No” may still provide a report. Raw Data indicates what kind(s) of genomic data is made available to participants upon their request, including FASTQ files of raw sequence reads, BAM files with aligned sequence reads and (g)VCF files of variants. CLIA/CAP indicates whether all elements of testing and return of results conformed to certification by Clinical Laboratory Improvements Amendment (CLIA) or College of American Pathologists (CAP). Projects with “No” may perform sub-components of the test, typically the sequencing itself, in a CLIA/CAP certified laboratory.

Project	Dates	Context	Individuals Sequenced to-Date	Platform	Laboratory Report with Signout	Results Returned			Sequencing Report Sent to Health Record	Raw Data Provided to Participants ^c^	CLIA/CAP
						Monogenic Disease	Common Disease	Pharmaco-Genomics			
PGP Harvard (with PGP Canada, PGP UK, Genome Austria) [2,39,40] *	2005–	Research	278	WGS	No	Filtered Variants w/Lit. Annotations			No	Variants	No
National Institute of Health (NIH) ClinSeq [41]	2006–	Research	1001	WES	If finding	X		X	Confirmed variants only	No	Conf ^b^
Baylor Young President’s Organization (YPO) [42] *	2010–2011	Research	81	WES	No	X			No	No	No
23andMe Exome Pilot [43,44]	2011–2012	Commercial	~150	WES	No	Filtered Variants			No	BAM, VCF	No
BWH ^d^/Harvard MedSeq Project [45,46,47] *	2011–	Research	60	WGS	Yes	X	X	X	Yes	FASTQ	Yes
Mount Sinai HealthSeq [48] *	2012–2015	Research	40	WGS	No	X	X	X	No	BAM, VCF	No
Mount Sinai PAPG [37]	2012–	Education	78	WGS	N/A	N/A			N/A	FASTQ	No
Illumina TruGenome™ Predisposition Screen including Understand Your Genome (UYG) [49] *	2012–	Commercial; Education	~850	WGS	Yes	X		X	At discretion of ordering MD	VCF	Yes
Baylor MD/PhD *	2013	Research	45	WES	Yes	X			No	No	Yes
Mayo “10 scientists” [50]	2012–2014	Research	10	WES	No	X			No	Yes	No
Stanford Genomic Medicine Application Pilot (GMAP) [51]	2013–2014	Research	12	WGS	? ^d^	X	X	X	No	?	No
Geisinger MyCode Community Health Initiative [52]	2007–	Research	~60,000	WES	If finding	X			Confirmed variants only	No	Conf ^b^
Institute for Systems Biology (ISB) Pioneer 100 [25,53] *	2014	Research	108	WGS	No	X	X	X	No	BAM, VCF	No
University of North Carolina (UNC) Genescreen [54]	2014–	Research	0	TGT ^a^	If finding	X			Yes	No	Conf ^b^
Baylor Miraca Adult Screening Exome [55]	2015–	Commercial	?	WES	Yes	X		X	At discretion of ordering MD	No	Yes
BWH/BCH ^d^/Harvard BabySeq Project [38] *	2015–	Research	30	WGS	Yes	X		X	Yes	FASTQ	Yes
Nevada Institute of Personalized Medicine *	2015–	Research	0	WES	Yes	X		X	No	BAM, VCF	No
Invitae Preventative Genetics Pilot Study *	2016–	Research; Commercial	0	TGT ^a^	Yes	X			Yes	No	Yes

^a^ Targeted (TGT) panel; ^b^ Confirmatory testing is performed in a CLIA/CAP-certified laboratory; the sequencing is not; ^c^ If requested by the participant; ^d^ Brigham and Women’s Hospital (BWH), Boston Children’s Hospital (BCH); ^d^ ‘?’ indicates unknown field.

**Table 2:** Categories of data collected by the PeopleSeq Consortium.

Background and Psychosocial	Knowledge and Perceptions	Medical, Behavioral and Economic Outcomes
Sociodemographics Health status and lifestyle Family medical history Psychological status Risk perceptions	Motivations and expectations Genomic knowledge Comprehension of results Perceived utility of PGS	Health and wellness behaviors Information seeking Sharing information Insurance-related behaviors Healthcare utilization
